# Surgical Outcome After Pancreatoduodenectomy for Duodenal Adenocarcinoma Compared with Other Periampullary Cancers: A Nationwide Audit Study

**DOI:** 10.1245/s10434-022-12701-y

**Published:** 2022-12-19

**Authors:** Jacob K. de Bakker, J. Annelie Suurmeijer, Jurgen G. J. Toennaer, Bert A. Bonsing, Olivier R. Busch, Casper H. van Eijck, Ignace H. de Hingh, Vincent E. de Meijer, I. Quintus Molenaar, Hjalmar C. van Santvoort, Martijn W. Stommel, Sebastiaan Festen, Erwin van der Harst, Gijs Patijn, Daan J. Lips, Marcel Den Dulk, Koop Bosscha, Marc G. Besselink, Geert Kazemier, for the Dutch Pancreatic Cancer Group

**Affiliations:** 1 Amsterdam UMC, Vrije Universiteit, Department of Surgery, Amsterdam, The Netherlands; 2 Cancer Center Amsterdam, Amsterdam, The Netherlands; 3 Amsterdam UMC, University of Amsterdam, Department of Surgery, Amsterdam, The Netherlands; 4 Department of Surgery, Leiden University Medical Center, Leiden, The Netherlands; 5 Department of Surgery, Erasmus University Medical Center, Rotterdam, The Netherlands; 6 Department of Surgery, Catharina Cancer Institute, Eindhoven, The Netherlands; 7 Department of Surgery, University of Groningen, University Medical Center Groningen, Groningen, The Netherlands; 8 Department of Surgery, Regional Academic Cancer Center Utrecht, St. Antonius Hospital and University Medical Center, Utrecht, The Netherlands; 9 Department of Surgery, Radboud University Medical Center, Nijmegen, The Netherlands; 10 Department of Surgery, OLVG, Amsterdam, The Netherlands; 11 Department of Surgery, Maasstadziekenhuis, Rotterdam, The Netherlands; 12 Department of Surgery, Isala Clinics, Zwolle, The Netherlands; 13 Department of Surgery, Medisch Spectrum Twente, Enschede, The Netherlands; 14 Department of Surgery, Maastricht University Medical Center, Maastricht, The Netherlands; 15 Department of Surgery, Jeroen Bosch ziekenhuis, Den Bosch, The Netherlands

## Abstract

**Background:**

Surgical outcome after pancreatoduodenectomy for duodenal adenocarcinoma could differ from pancreatoduodenectomy for other cancers, but large multicenter series are lacking. This study aimed to determine surgical outcome in patients after pancreatoduodenectomy for duodenal adenocarcinoma, compared with other periampullary cancers, in a nationwide multicenter cohort.

**Methods:**

After pancreatoduodenectomy for cancer between 2014 and 2019, consecutive patients were included from the nationwide, mandatory Dutch Pancreatic Cancer Audit. Patients were stratified by diagnosis. Baseline, treatment characteristics, and postoperative outcome were compared between groups. The association between diagnosis and major complications (Clavien–Dindo grade III or higher) was assessed via multivariable regression analysis.

**Results:**

Overall, 3113 patients, after pancreatoduodenectomy for cancer, were included in this study: 264 (8.5%) patients with duodenal adenocarcinomas and 2849 (91.5%) with other cancers. After pancreatoduodenectomy for duodenal adenocarcinoma, patients had higher rates of major complications (42.8% vs. 28.6%; *p* < 0.001), postoperative pancreatic fistula (International Study Group of Pancreatic Surgery [ISGPS] grade B/C; 23.1% vs. 13.4%; *p* < 0.001), complication-related intensive care admission (14.3% vs. 10.3%; *p* = 0.046), re-interventions (39.8% vs. 26.6%; *p* < 0.001), in-hospital mortality (5.7% vs. 3.1%; *p* = 0.025), and longer hospital stay (15 days vs. 11 days; *p* < 0.001) compared with pancreatoduodenectomy for other cancers. In multivariable analysis, duodenal adenocarcinoma was independently associated with major complications (odds ratio 1.14, 95% confidence interval 1.03–1.27; *p* = 0.011).

**Conclusion:**

Pancreatoduodenectomy for duodenal adenocarcinoma is associated with higher rates of major complications, pancreatic fistula, re-interventions, and in-hospital mortality compared with patients undergoing pancreatoduodenectomy for other cancers. These findings should be considered in patient counseling and postoperative management.

**Supplementary Information:**

The online version contains supplementary material available at 10.1245/s10434-022-12701-y.

Duodenal adenocarcinoma is a rare disease with a rising incidence.^1^ Locoregional duodenal and other periampullary cancers are primarily treated by pancreatoduodenectomy and, potentially, adjuvant chemotherapy.^1–3^ Tumor morphology of duodenal adenocarcinoma demonstrates an intestinal-type differentiation, resembling colorectal cancer, while most other periampullary cancers have a pancreaticobiliary-type differentiation.^4^ Better overall survival has been reported for patients with duodenal adenocarcinoma compared with patients with other periampullary cancers.^4,5^

Mortality after pancreatoduodenectomy has decreased over the last decades. Reported in-hospital mortality rates are < 5% in specialized centers,^6,7^ which is partially due to centralization of the operation, with pancreatoduodenectomy being increasingly performed in high-volume centers,^8^ and better management of major complications such as postoperative pancreatic fistula (POPF) and their sequalae, abdominal sepsis, and post-pancreatectomy hemorrhage (PPH).^9,10^ While mortality rates for pancreatoduodenectomy have decreased considerably, postoperative morbidity rates can still reach up to 67%.^11–13^

In surgical series on pancreatoduodenectomy, outcomes for patients with duodenal adenocarcinoma are often combined with other periampullary cancers because of their low incidence.^14–16^ This results in limited knowledge of postoperative mortality and complication rates after pancreatoduodenectomy for duodenal adenocarcinoma. However, adequate identification of perioperative risk factors is pivotal for clinicians to better select patients with duodenal adenocarcinoma for curative resection and adequately counsel patients. The aim of this multicenter, nationwide audit-based study was to determine the postoperative morbidity and mortality after pancreatoduodenectomy for duodenal adenocarcinoma compared with other periampullary cancers.

## Methods

After pancreatoduodenectomy for duodenal adenocarcinoma and other periampullary cancers (pancreatic ductal adenocarcinoma, distal cholangiocarcinoma, and ampullary cancer) between 2014 and 2019, consecutive patients were included from the nationwide mandatory Dutch Pancreatic Cancer Audit (DPCA). This audit covers all pancreatic resections and demonstrates high accuracy and case ascertainment.^17^ In this study, only patients with adenocarcinoma were included based on final postoperative histopathology. The duodenal adenocarcinoma group included patients with a final diagnosis of duodenal adenocarcinoma, which did not include (intestinal type) ampullary carcinoma. Neither informed consent nor ethical approval for this study were required since the data are anonymously registered. Complication rates, hospital stay, and mortality after pancreatoduodenectomy were compared for duodenal adenocarcinoma and other periampullary cancers.

### Definitions

Postoperative complications were classified according to the Clavien–Dindo classification, of which only complications with at least grade IIIa were included (i.e., major complications). Pancreatic surgery-specific complications were scored according to the International Study Group of Pancreatic Surgery (ISGPS) definitions for POPF, post-pancreatectomy hemorrhage, delayed gastric emptying (DGE), and bile leakage.^18–21^ Only clinically relevant complications, defined as grade B/C, were included.

### Endpoints

The primary endpoint was the rate of major complications (Clavien–Dindo grade III or higher), while secondary endpoints included length of stay, in-hospital mortality rates, and pancreatic-specific complications.

### Statistical Analysis

Normally distributed continuous data were presented as means with standard deviations (SDs) and were compared using Student’s *t*-test. Non-normally distributed continuous data were expressed as medians with interquartile ranges (IQR) and assessed using the Mann–Whitney U test. Categorical data were presented in frequencies with percentages and analyzed using the Chi-square test. Multivariable complete-case logistic regression analyses, adjusted for all relevant confounders based on literature and expert opinion, was performed to assess the association between diagnosis and major complications (Clavien–Dindo grade III or higher). Confounders were patient characteristics (sex, age, body mass index [BMI], preoperative weight loss, and American Society of Anesthesiologists [ASA] score), perioperative care (neoadjuvant treatment, preoperative biliary drainage, perioperative use of octreotide), and intraoperative findings (pancreatic texture and diameter of the pancreatic duct).

## Results

### Baseline Characteristics

Overall, 3113 consecutive patients, after pancreatoduodenectomy for cancer, were included: 264 (9%) patients with duodenal adenocarcinomas and 2849 (91%) patients with other periampullary cancers, including 1753 (56%) patients with pancreas ductal adenocarcinomas, 546 (17%) patients with distal cholangiocarcinomas, and 550 (18%) patients with ampullary cancers. Baseline characteristics are summarized in Table 1. Patient characteristics (e.g., sex and age) did not differ between duodenal adenocarcinomas and other periampullary cancers. After pancreatoduodenectomy for duodenal adenocarcinoma, patients had less preoperative weight loss (4 vs. 5 kg; *p* = 0.018) and less often underwent preoperative biliary drainage (14% vs. 64%; *p* < 0.001).

**Table 1:** Patient and perioperative characteristics after pancreatoduodenectomy

	Overall [*n* = 3113]	Duodenal adenocarcinoma [*n* = 264]	Other periampullary cancers [*n* = 2849]	*p* value
Female sex	1370 (44)	116 (44)	1254 (44)	0.981
Age, years [median (IQR)]	69.0 (62.0–75.0)	68.0 (60.8–74.0)	69.0 (62.0–75.0)	0.085
BMI [median (IQR)]	24.0 (22.0–27.0)	24.0 (22.0–28.0)	24.0 (22.0–27.0)	0.562
Preoperative weight loss, kg [median (IQR)]	5.0 (2.0–8.0)	4.0 (0.0–9.0)	5.0 (2.0–8.0)	0.023
ASA score				0.618
1	333 (11)	29 (11)	304 (11)	
2	1932 (63)	156 (60)	1776 (63)	
≥ 3	816 (26)	75 (29)	741 (26)	
Charlson Comorbidity Index				0.025
0	1536 (49)	131 (50)	1405 (49)	
1	699 (22)	44 (17)	655 (23)	
≥ 2	878 (28)	89 (34)	789 (28)	
Biliary drainage	1862 (62)	37 (15)	1831 (66)	< 0.001
Neoadjuvant therapy	221 (7.3)	6 (2.4)	215 (7.8)	0.002
Perioperative octreotide	1899 (62)	176 (67)	1723 (60)	0.030
Type of surgery				0.114
Open	2577 (85)	229 (88)	2348 (84)	
Laparoscopic	216 (7.1)	18 (6.9)	198 (7.1)	
Robot-assisted	256 (8.4)	13 (5.0)	243 (8.7)	
Pancreatic texture				< 0.001
Soft or normal	1643 (58)	203 (84)	1440 (56)	
Hard or fibrotic	1189 (42)	38 (16)	1151 (44)	
Pancreatic duct diameter, mm [median (IQR)]	4.0 (3.0–6.0)	3.0 (2.0–4.0)	4.0 (3.0–6.0)	< 0.001

Data are expressed as *n* (%) unless otherwise specified

*BMI* body mass index, *ASA* American Society of Anesthesiologists, *IQR* interquartile range

Pearson's Chi-square test; Wilcoxon rank-sum test; Fisher's exact test

### Surgical Outcome

Postoperative complications and mortality are shown in Table 2. In total, 929 (29.8%) patients experienced major complications, which were more often seen after pancreatoduodenectomy for duodenal adenocarcinoma (42.8% vs. 28.6%; *p* < 0.001), compared with other periampullary cancers.

**Table 2:** Surgical outcome after pancreatoduodenectomy

	Overall [*n* = 3113]	Duodenal adenocarcinoma [*n* = 264]	Other periampullary cancers [*n* = 2849]	*p* value^a^
Major complications^b^	901 (30)	109 (43)	792 (29)	< 0.001
Postoperative pancreatic fistula^c^	443 (14)	61 (23)	382 (13)	< 0.001
Post-pancreatectomy hemorrhage^c^	234 (7.6)	24 (9.3)	210 (7.5)	0.298
Delayed gastric emptying^c^	561 (18)	65 (25)	496 (18)	0.002
Bile leakage^c^	128 (4.2)	22 (8.4)	106 (3.8)	< 0.001
Postoperative interventions	863 (28)	105 (40)	758 (27)	< 0.001
Intensive care admission	328 (11)	37 (14)	291 (10)	0.043
Length of stay, days [median (IQR)]	12.0 (8.0–18.0)	15.0 (9.0–23.8)	11.0 (8.0–17.0)	< 0.001
Readmission	467 (16)	45 (18)	422 (16)	0.242
In-hospital mortality	106 (3.4)	15 (5.7)	91 (3.2)	0.033

Data are expressed as *n* (%) unless otherwise specified

^a^Pearson's Chi-square test; Fisher's exact test

^b^Clavien–Dindo grade III or higher

^c^Grade B/C according to the International Study Group of Pancreatic Surgery/International Study Group of Liver Surgery (ISGPS/ISGLS) criteria

In-hospital mortality was higher in patients with duodenal adenocarcinoma (5.7% vs. 3.1%; *p* = 0.025). Re-intervention was more common in patients with duodenal adenocarcinoma (39.8% vs. 26.6%; *p* < 0.001), especially regarding radiologic drainage (28.3% vs. 17.6%; *p* < 0.001), and complications leading to intensive care admission occurred more often in patients with duodenal adenocarcinoma (14.3% vs. 10.3%; *p* = 0.046). Median length of hospital stay was longer for patients with duodenal adenocarcinoma in comparison with other periampullary cancers (15 days [IQR 9–24] vs. 11 days [IQR 8–17]; *p* < 0.001). Subgroup analysis of postoperative outcome stratified by diagnosis is shown in electronic supplementary Table 1.

### Multivariable Analysis for Major Complications

Overall, 1881 patients were included in the multivariable logistic regression analysis for the development of major complications (Table 3). Multivariable analysis identified male sex (odds ratio [OR] 1.31, 95% confidence interval [CI] 1.04–1.64; *p* = 0.022), ASA score of III or higher (OR 1.64, 95% CI 1.05–2.56; *p* = 0.031), pancreatic texture (OR 0.65, 95% CI 0.51–0.83; *p* = 0.001), pancreatic duct diameter (OR 0.93, 95% CI 0.89–0.97; *p* < 0.001), and duodenal adenocarcinoma (OR 1.14, 95% CI 1.03–1.27; *p* = 0.011) as independent factors influencing the development of major complications.

**Table 3:** Association between preoperative variables and major complications in a multivariable regression analysis

	OR	95% CI	*p* value
Age	1.000	0.988–1.012	0.998
Male sex	1.306	1.039–1.641	0.022
BMI	1.016	0.989–1.043	0.245
Preoperative weight loss	0.984	0.961–1.007	0.169
ASA score			
ASA 1	Reference		
ASA 2	1.087	0.722–1.637	0.715
ASA ≥ 3	1.637	1.045–2.564	0.031
Perioperative octreotide	1.115	0.881–1.412	0.365
Preoperative biliary drainage	0.922	0.772–1.264	0.988
Pancreatic texture (hard/firm)	0.651	0.511–0.830	0.001
Neoadjuvant therapy	1.069	0.945–1.069	0.867
Pancreatic duct diameter	0.930	0.893–0.967	< 0.001
Diagnosis (duodenal adenocarcinoma)	1.142	1.031–1.266	0.011

*OR* odds ratio, *CI* confidence interval, *BMI* body mass index, *ASA* American Society of Anesthesiologists

## Discussion

This first multicenter nationwide study on surgical outcome after pancreatoduodenectomy for duodenal adenocarcinoma found more major complications, more re-interventions, longer postoperative stay, and higher mortality compared with pancreatoduodenectomy for other periampullary cancers.

Only a few, mostly small, monocenter studies reported on surgical outcome after pancreatoduodenectomy for duodenal adenocarcinoma and compared these with outcomes for other periampullary tumors.^14,15,22–27^ An overview of these studies, based on a systematic search, is shown in Table 4. The reported rates of major complications after PD for duodenal adenocarcinoma varied between 18 and 24%.^14,15,27^ Some of these studies included only patients with duodenal adenocarcinoma, whereas others performed a subgroup analysis within a larger cohort. None of these studies included all endpoints reported in the current study. The higher rate of major complications found in the present study may be related to the prospective nature of the DPCA. Moreover, the current study may better reflect more daily clinical practice than monocenter studies.

**Table 4:** Studies on pancreatoduodenectomy for duodenal adenocarcinoma

Author	Year	Mono- vs. multicenter	*N*	POPF B/C (%)	PPH B/C (%)	DGE B/C (%)	In-hospital mortality (%)	CD grade III or higher	Hospital stay
Bakaeen et al.^22^	2000	Mono	45	16 (35)^b^	NR	NR	1 (2)	NR	NR
He et al.^23^	2014	Mono	158	17 (11)^b^	NR	16 (10)	NR	NR	NR
Lee et al.^25^	2014	Mono	44	11 (25)^b^	NR	1 (2)	0 (0)	NR	NR
Shamali et al.^26^	2016	Mono	40^a^	10 (25)	4 (10)	5 (13)	2 (5)	NR	15
Lee et al.^24^	2016	Multi	38^a^	9 (24)	NR	NR	3 (10)	NR	NR
Bourgouin et al.^14^	2017	Mono	25	6 (24)	4 (16)	4 (16)	1 (4)	6 (24%)	22
Wiltberger et al.^27^	2018	Mono	46	8 (17)	3 (7)	NR	4 (9)	11 (24%)	19
Jung et al.^15^	2020	Mono	131	14 (11)	NR	NR	2 (2)	24 (18%)	16
Wang et al.^16^	2021	Mono	92	9 (10)	6 (7)	9 (10)	0 (0)	16 (17%)	NR
De Bakker et al.	Current	Multi	264	61 (23)	24 (9)	65 (25)	15 (6)	113 (43%)	15 (9–24)

*N* number, *POPF* postoperative pancreatic fistula, *PPH* post-pancreatectomy hemorrhage, *DGE* delayed gastric emptying, *CD* Clavien–Dindo, *NR* not reported

^a^Adenoma also included

^b^No differentiation in B/C grade

Based on a PubMed search: “pancreaticoduodenectomy AND adenocarcinoma AND complications AND (duodenum OR duodenal)”

Multivariable analysis confirmed the positive correlation between high ASA score, soft pancreatic texture, small pancreatic duct diameter, and the occurrence of major complications.^28,29^ It is widely recognized that soft pancreatic texture and small diameter of the pancreatic duct are associated with POPF; however, this study confirms that both are also associated with major complications. Patients with duodenal adenocarcinoma usually have both these risk factors, which poses them at greater risk for complications.^15,26^ However, in the present analysis, even after adjustment for pancreatic texture and duct diameter, duodenal adenocarcinoma as an indication for pancreatoduodenectomy remained an independent risk factor for postoperative complications.

In the current study, somatostatin analogs (octreotide or pasireotide) were used more often in patients with duodenal adenocarcinoma (67% vs. 60%; *p* = 0.03); however, the value of these agents in preventing POPF in all patients undergoing a pancreatoduodenectomy remains debated.^30,31^ Based on our results, no benefit of somatostatin use was observed in patients after pancreatoduodenectomy for duodenal adenocarcinoma.

Neoadjuvant or induction chemotherapy, radiotherapy, or a combination has been more frequently provided for patients with pancreatic cancer over the last decade, which also affects patients included in the current analysis. Neoadjuvant therapy could impact the texture of pancreatic tissue, which has been associated with reduced rates of POPF.^32^

The results of the present study should be interpreted considering several limitations. First, there was potential information bias regarding the lack of information on patient’s preoperative nutritional status as no data on nutritional status are available in the nationwide, prospectively recorded DPCA. Preoperative nutrition status in pancreatic surgery has been recognized as a significant variable in postoperative mortality and morbidity.^33^ Malignancies such as PDAC are widely recognized to be associated with cachexia and malnutrition.^34^ Jaundice, exocrine insufficiency, and weight loss are the most common preoperative findings noted in patients with PDAC.^35,36^ A gastric outlet obstruction caused by duodenal adenocarcinoma can lead to cachexia and malnutrition, which further impairs nutrition status. Second, although all complications are registered during hospital admission or within 30 days postoperatively, no information was available on the exact timing in which complications in the present study population were diagnosed and/or treated. Third, data on bile duct diameter were not available; however, it seems likely that the higher rate of bile leak is related to less-dilated bile ducts in patients with duodenal adenocarcinoma compared with other patients undergoing pancreatoduodenectomy. Fourth, during the majority of the study period, the Dutch hospitals had no uniform postoperative algorithm for detecting and treating complications. Such a protocol has recently been implemented during the nationwide PORSCH trial, which included patients with duodenal adenocarcinoma and reduced mortality after pancreatic surgery.^37^ Fifth, no data on adjuvant chemotherapy were available. Major morbidity could influence the rate of adjuvant chemotherapy, for instance, in patients with positive nodal status. Efforts are underway to couple the DPCA to the Dutch cancer registry, allowing for such analyses.

Ways to reduce the relatively high rates of major complications after pancreatoduodenectomy for duodenal adenocarcinoma should be the subject of further investigation. Preventive measurements such as preoperative prehabilitation could potentially contribute in this manner.^38^ The role of somatostatin analogs in the prevention of POPF remains unclear.^30,39^ An intervention to alter the pancreatic texture using stereotactic radiotherapy is currently being investigated in the multicenter phase II FIBROPANC trial (NL9299).

## Conclusion

Pancreatoduodenectomy for duodenal adenocarcinoma is associated with higher rates of major complications and in-hospital mortality compared with patients undergoing pancreatoduodenectomy for other periampullary cancers. This finding is relevant when counseling patients with duodenal adenocarcinoma undergoing pancreatoduodenectomy and optimizing postoperative treatment strategies.

## Supplementary Information

Below is the link to the electronic supplementary material.

Supplementary file1 (DOCX 16 kb)

## Data Availability

The authors had complete access to the study data that support this publication. All relevant data are within the paper and its electronic supplementary files. Data, analytic methods, and study materials can be made available to other researchers on reasonable request.
