# Supplementary material for: Surgical Outcome After Pancreatoduodenectomy for Duodenal Adenocarcinoma Compared with Other Periampullary Cancers: A Nationwide Audit Study
Source: Ann Surg Oncol. 2022 Dec 19;30(4):2448–55. doi: 10.1245/s10434-022-12701-y (PMC10027630; doi:10.1245/s10434-022-12701-y)
Supplement: Supplementary file 1 — Supplementary file1 (DOCX 16 kb) [file 10434_2022_12701_MOESM1_ESM.docx]

**Supplementary table 1. Postoperative outcome after pancreatoduodenectomy stratified by diagnosis**

|  | Overall  N = 3,113 | Pancreatic adenocarcinoma  N = 1,753 | Cholangiocarcinoma  N = 546 | Ampullary cancer  N = 550 | Duodenal adenocarcinoma  N = 264 |
| --- | --- | --- | --- | --- | --- |
| Major complications^#^ | 901 (30%) | 407 (24%) | 204 (39%) | 181 (34%) | 109 (43%) |
| Missing | 90 | 44 | 22 | 16 | 8 |
| Postoperative pancreatic fistula* | 443 (14%) | 137 (7.9%) | 132 (24%) | 113 (21%) | 61 (23%) |
| Missing | 17 | 9 | 3 | 5 | 0 |
| PPH* | 234 (7.6%) | 108 (6.2%) | 42 (7.8%) | 60 (11%) | 24 (9.3%) |
| Missing | 44 | 23 | 9 | 7 | 5 |
| Delayed gastric emptying* | 561 (18%) | 263 (15%) | 123 (23%) | 110 (20%) | 65 (25%) |
| Missing | 35 | 11 | 9 | 8 | 7 |
| Bile leakage* | 128 (4.2%) | 50 (2.9%) | 31 (5.8%) | 25 (4.6%) | 22 (8.4%) |
| Missing | 32 | 14 | 7 | 9 | 2 |
| Postoperative interventions | 863 (28%) | 385 (22%) | 199 (37%) | 174 (32%) | 105 (40%) |
| Missing | 60 | 30 | 15 | 12 | 3 |
| Intensive care admission | 328 (11%) | 153 (8.8%) | 69 (13%) | 69 (13%) | 37 (14%) |
| Missing | 41 | 22 | 6 | 7 | 6 |
| Length of stay (days) | 12.0 (8.0 - 18.0) | 11.0 (8.0 - 16.0) | 13.0 (9.0 - 20.0) | 12.0 (8.0 - 19.0) | 15.0 (9.0 - 24.0) |
| Missing | 34 | 23 | 3 | 5 | 3 |
| Readmission | 467 (15%) | 268 (16%) | 82 (15%) | 72 (13%) | 45 (17%) |
| Missing | 49 | 25 | 13 | 7 | 4 |
| In-hospital mortality | 103 (3.3%) | 49 (2.8%) | 19 (3.5%) | 20 (3.6%) | 15 (5.7%) |
| Missing | 6 | 4 | 1 | 1 | 0 |
| n (%); Median (IQR) PPH: Post Pancreatectomy Hemorrhage  #Complications Clavien Dindo ≥III  *All grade B/C according to ISGPG/ISGLG criteria | | | | | |
